# Constipation

**Published:** 1890-04-26

**Authors:** 


### CONSTIPATION.

Dr. Thomas, Consulting Physician to the Sheffield Hospital, writes, in the *Lancet*, on the utility of massage in the treatment of diseases of some of the abdominal organs. He does not think the efficacy of massage is sufficiently appreciated in some of those diseases. He therefore relates two typical cases out of a number which have come under his notice as illustrative of the benefit to be derived from the treatment. The first case was one of very pressing constipation, with large swelling of the bowels, supposed to be caused by a malignant obstruction. On examination the bowels were found, "I may almost say craggy." Injections were ordered, with careful rubbing, which afforded complete relief after some days. Secondly, a female had suffered years ago from a severe attack of typhlitis, and now had costive bowels, swelling, and slight tenderness in the right iliac region. Evidently the colon was distended by a large fæcal mass, from the cæcum to the right angle. Injections were given, and rubbing only brought to bear on the mass at the upper third as the cæcum was tender. But as the scybalæ became dislodged the tenderness was lessened, and ultimately the whole mass was removed. Dr. Thomas observes that it is evidently intended by Nature that the movement of the intestinal tube should be aided by active concurrence of the muscles of the abdomen, and it is equally evident that the liver and gall bladder should be pressed upon. Exercise secures this result, but while many will drink aperient waters, and take pills to secure action of the bowels, they neglect the essential of exercise, which tends to induce a healthy state of the abdominal viscera. Whenever a patient goes to Dr. Thomas, and gives a previous history of trouble in the cæcum, colon, or sigmoid flexure, and complains of constipation, flatulence, pain, &c., Dr. Thomas generally finds fæcal accumulation, which if neglected would probably terminate in inflammatory mischief. The condition is soon relieved by injections and careful rubbing. But massage is a potent agent for good or evil. If the bowel is acutely inflamed much mischief ensues. But Dr. Thomas observes: "I may say in cases of typhlitis with accumulation in the ascending colon, more can often be done than by prescribing morphia, diet, and rest," and thus letting the cause of all the mischief remain and irritate.

